# Variabilities in the stroking parameters during short course 50 m time trials in all four competitive swimming strokes

**DOI:** 10.1038/s41598-025-08519-9

**Published:** 2025-07-02

**Authors:** Tomohiro Gonjo, Santiago Veiga, Francisco Hermosilla-Perona, Bjørn Harald Olstad

**Affiliations:** 1 School of Energy, Geoscience, Infrastructure and Society, Institute for Life and Earth Sciences, Heriot-Watt University, Edinburgh, UK; 2 Department of Physical Performance, Norwegian School of Sport Sciences, Oslo, Norway; 3 Sports Department, Universidad Politécnica de Madrid, Madrid, Spain; 4 Department of Physical Activity and Sports Science, Alfonso X El Sabio University, Madrid, Spain; 5 Facultad de Ciencias de la Vida y la Naturaleza, Nebrija University, Madrid, Spain

**Keywords:** Functional variability, Aquatic locomotion, Butterfly, Backstroke, Breaststroke, Front crawl, Sprint swimming, Scientific data, Statistics, Data acquisition, Data processing, Human behaviour

## Abstract

The purpose of this study was to identify intra- and inter-individual variabilities during short course 50 m sprints. Swimming velocity (*SV*), stroke frequency (*SF*), and stroke length (*SL*) for each stroke cycle in 189 male and 160 female swimmers’ 50 m time trials (with their specialised stroke) were analysed. The inter-individual variability for each kinematic variable was analysed using the inter-individual standard deviation of the Gaussian Process regression. Intra-participant variability was analysed using k-means clustering with kinematic data extracted from the first, mid-, and last strokes. In all strokes and both sexes, swimmers showed large inter-individual kinematic variabilities at the first and last strokes, which justified the need to separate these strokes from the clean-swimming segment in race analyses. Intra-individual kinematic patterns were categorised into four clusters with different within-lap *SV* patterns. Particularly, many front crawl and backstroke swimmers showed a faster velocity in mid-pool than in the transition, while many butterfly swimmers showed the fastest *SV* in the transition. This might suggest a greater difficulty in the transition technique in alternating strokes than in butterfly. Race analyses should focus on not only the overall trend but also individual variabilities to investigate the swimmers’ behaviour during swimming races.

## Introduction

Competitive swimming races consist of either or all of the four swimming strokes (butterfly, backstroke, breaststroke, and front crawl), and the performance of a race depends on the time and distance spent in the start, turn, and surface swimming segments^1,2^. These segments are defined according to the swimmer’s cyclical (surface swimming) or acyclic (start and turn) movements. The current World Aquatics regulations limit the maximum distance for underwater swimming after diving off the starting block or pushing off the turning wall to 15 m (in all strokes except breaststroke), and therefore, start and turn times are usually evaluated at 15 m. The rest of the lap corresponds to the surface swimming, also referred to as the clean-swimming segment^3^. Depending on the skill level of the swimmer, the race segment definitions can be adapted using different definitions of the start or turn(s)^4^or individualised distance segments can be defined from the exact point of emersion of each swimmer^5^. In addition, the last 5–7.5 m of each lap has often been considered as the finish (in the last lap), or a part of the turn segment (turn-in segment) to account for hypothetical stroke modifications when swimmers approach the wall^6^. However, the rationales behind these segmental definitions, particularly the distance of the turn-in and finish segments, have been somewhat unclear.

For the clean swimming segment, race analysts typically capture a range of distance from each swimming lap to provide quantitative feedback to coaches and swimmers^3^. For example, mid-pool segments, such as between 15 m and 35 m of a long course pool^7,8^, between 10 and 20 m (15 m and 20 m in the first lap) in a short course pool^7,8^, or three to five selected stroke cycles^9^, have been used to calculate the average swimming speed. Although very practical and not time-consuming during major competitions (especially for feedback between rounds, such as between heats and finals), this procedure cannot describe intra-lap variations that could be expected in swimming kinematics. In fact, previous data from the World Swimming Championships showed changes in the swimming parameters from the first to the middle swimming strokes of 100 m races^10^ which were related to higher velocities in the start and turn segments compared to clean-swimming. In other words, a positive effect of the initial speed on swimming speed at the beginning of the swimming laps could be expected if swimmers optimised their starting and turning movements^11^ which should be considered when examining the different race segments^12^.

A certain degree of variation in cyclic movements, known as functional variability for dynamics systems theories, can help performers adapt to the constantly changing constraints^13^. For competitive swimmers, changes in the task constraints are typically associated with the aforementioned influence of the start and turn segments on the swimmer’s clean-swimming speed, changes in the environmental constraints, such as waves and water turbulence or the wave effect from other competitors^14^, and fatigue during the race^15^. All these constraints could alter the swimmer’s behaviour within the swimming lap. Consequently, investigating the levels of inter- or intra-individual variability in stroking parameters could provide useful information on how swimmers manage the different constraints of the race^16^. Previous research has described a higher level of intra-lap variability in stroke frequency in the first lap of 100 m front crawl events but less variation in the stroke length and velocity compared to the second lap^17^. In 200 m front crawl events, competitive swimmers showed greater levels of intra-lap variability in the first compared to the second lap^14^. However, all the evidence on the real competitive environment could be influenced by many external factors, such as the relative position of each swimmer compared to the rest of the competitors^18^. Therefore, investigating the variabilities in individual time-trial conditions could be useful.

In recent years, new technologies have come into practice to provide in-depth information about swimmers’ performance in individual time trials and to overcome some of the limitations of traditional race analysis procedures. For example, a previous study described the start and turn parameters of butterfly competition swimmers in detail, including the kinematic characteristics of forward speed in the underwater swimming segments^19^. In relation to surface swimming, another study modelled the stroke parameters (stroke by stroke) of elite and non-elite swimmers within and between laps in breaststroke races^20^. Stroke-to-stroke information is considered a non-linear complex system with different stroke models to achieve the same goal^21^. Nevertheless, in the literature, such information is lacking for the different official strokes and for efforts swum in a 25 m short course pool, where the influence of the start and turn segments on overall performance is even greater^22^.

Therefore, the aim of the present study was to identify intra- and inter-individual variabilities of competitive swimmers during short course 50 m time trials. It was hypothesised that inter-individual variability in swimming would increase at the beginning and end of swimming laps, regardless of stroke, and the majority of swimmers would show faster swimming velocity at the beginning of the lap compared with the rest for the intra-individual variability.

## Materials and methods

### Participants

A total of 349 competitive swimmers (189 males and 160 females) with a minimum World Aquatics (WA) point of 450 were recruited for the study. The threshold of the WA point was determined according to the literature^23^ which suggested that this point was the threshold for competitive swimmers at the regional swimming level (Level 4). They were divided into eight groups according to their sex and speciality stroke (Table 1). The procedure, risks, and benefits of the study were reviewed and approved by the local Ethical Committee and the National Data Protection Agency for Research in accordance with the Declaration of Helsinki. All participants (or legal guardians for minors) received detailed verbal and written explanations of the study, and written informed consent was obtained from each of them.

**Table 1:** Demographics (mean ± standard deviation) of the participants.

		Age (years)	Height (m)	Mass (kg)	WA points
Butterfly	Male (*N* = 42)	19.18 ± 4.14	1.85 ± 0.06	78.55 ± 8.80	629.26 ± 94.89
	Female (*N* = 28)	17.92 ± 1.72	1.71 ± 0.06	64.41 ± 7.76	620.07 ± 55.25
Backstroke	Male (*N* = 28)	19.06 ± 3.42	1.87 ± 0.07	78.75 ± 10.75	590.21 ± 85.09
	Female (*N* = 33)	17.44 ± 1.69	1.71 ± 0.07	64.66 ± 8.72	612.09 ± 73.16
Breaststroke	Male (*N* = 36)	19.40 ± 4.22	1.85 ± 0.07	78.45 ± 10.73	641.14 ± 96.88
	Female (*N* = 30)	18.08 ± 3.12	1.69 ± 0.06	62.83 ± 5.28	619.83 ± 73.34
Front crawl	Male (*N* = 83)	18.94 ± 4.77	1.85 ± 0.06	76.90 ± 9.47	621.96 ± 82.92
	Female (*N* = 69)	17.18 ± 2.48	1.71 ± 0.07	65.53 ± 8.09	598.04 ± 65.55

### Data collection

Testing was conducted in a 25 m indoor swimming pool with water and air temperatures of 27 Cº and 28 Cº, respectively. The swimmers were instructed to perform their individual warm-up procedures on land and in water, as they usually do in competitions, for 45–60 min. After the warm-up, they changed into their competition swimsuits and performed a 50 m time trial from a competitive start with their specialised stroke, aiming to achieve the fastest time possible. The trial was recorded using a race analysis system (AIMsys Sweden AB, Lund, Sweden) consisting of 11 stationary digital video cameras (of which one camera was used solely for feedback purposes). Five cameras were mounted approximately 5 m above the water surface, and five were mounted below the water surface along with the 25 m pool. The underwater cameras were located 0.70 m beneath the water surface perpendicular to the swimming direction (5 m apart between the cameras). All cameras were synchronised, and the recording was conducted with a 50 Hz sampling frequency and a camera resolution of 1080p. The system was also synchronised with an electric timing system that timed the 50 m record.

The system was pre-calibrated in the manner described in the literature^8,24^ which yielded a mean calibration error of 0.025°angular error and 0.006 m linear error. The system was also equipped with an image recognition algorithm that tracked the swimmer’s head (with a yellow silicon cap), as well as the timing of the first backward movement of the hand in relation to the swimmer’s body. Using this set of information, the system further generated the time-series forward velocity data of the head (*v*), stroke frequency (*SF*), and stroke length (*SL*) for all strokes throughout the trials. The mean swimming velocity data for each stroke cycle (*SV*) were also calculated from the time-series *v* data.

### Data analysis

Each kinematic variable was expressed as the percentage of the individual intra-lap mean value for further analyses. In addition, using head position data for the beginning of each stroke cycle, *SV*, *SF*, and *SL* data were expressed as the data against the normalised distance from the first stroke (0%) to the last stroke of the lap (100%). The inter-individual trend of each kinematic data for each lap was obtained using a non-parametric regression model: the locally-weighted scatterplot smoothing (LOWESS) model with a moving window size of 30% time. For inter-individual variability analysis, kinematic data for each participant for each lap were converted into an individual regression model using the Gaussian Process (GP) regression. The inter-participant variability was then expressed as the inter-participant standard deviation of the models at each normalised distance point.

**Fig. 1:**
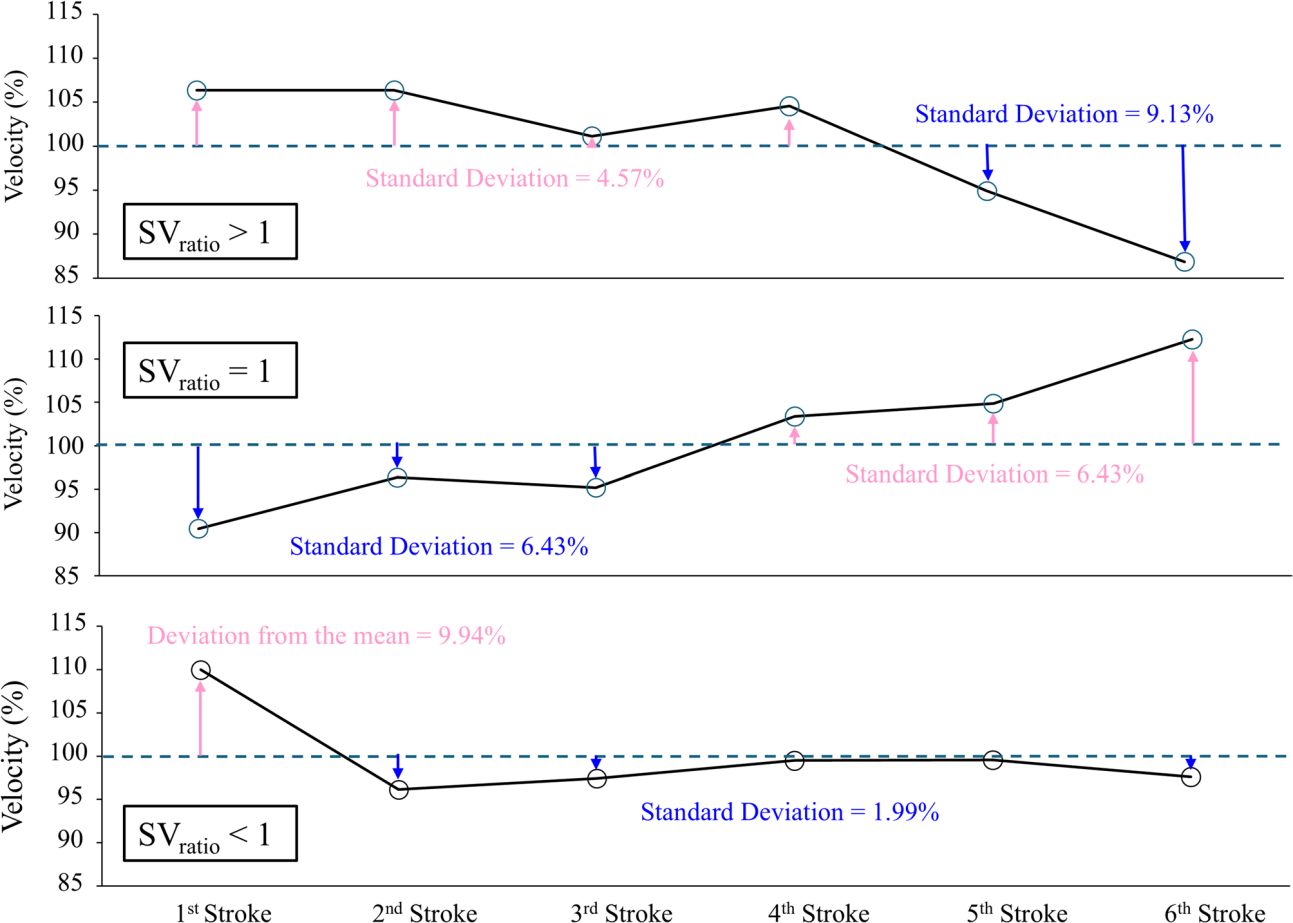
Graphical examples of the ratio of the number of strokes over 100% (above the intra-lap mean) to that under 100% (below the intra-lap mean).

Intra-individual variability of the kinematic information was analysed using an unsupervised machine-learning approach. First, the kinematic data from the first-stroke, mid-pool stroke (mid-stroke), and last-stroke were extracted from each participant and lap. If the stroke count was an odd number, the mid-stroke was the middle value in the sequence of data for the lap, while if the stroke count was an even number, the mid-stroke was calculated as the mean of the two middle values. Selecting these three strokes was necessary to standardise the number of data for all participants, whereas this approach would lose information from the other strokes. Therefore, for each participant/lap/variable, the ratio of the number of strokes above the intra-lap mean value to the number of strokes below the intra-lap mean value was obtained (*SF*_*ratio*_, *SL*_*ratio*_, and *SV*_*ratio*_, respectively). When the ratio is greater than 1, it shows that the swimmer achieved a higher number of strokes above the intra-lap mean than the number of strokes below the mean (Fig. 1). All of these variables (*SF*, *SL*, and *SV* for the first-, mid-, and last-stroke and *SF*_*ratio*_, *SL*_*ratio*_, and *SV*_*ratio*_) for both laps and genders of all strokes were put into k-means cluster analysis to identify different combinations of kinematic variables and patterns using NbClust version 3.0.1^25^ with RStudio 2023.6.0. For the cluster analysis, the Euclidian distance was used to compute the dissimilarity matrix, and the number of clusters was determined based on 30 different validation algorithms using the NbClust package. The Fisher information was also calculated for all variables included in the analysis to assess the impact of each measure on the clustering. Fisher information is the ratio between the sum of inter-cluster and intra-cluster distances and can be computed for each variable as;

$$\:FI=\frac{{\sum\:}_{g}{N}_{g}\cdot \:{d}^{2}\left({\mu\:}_{g},\stackrel{-}{X}\right)}{{\sum\:}_{g}{\sum\:}_{i\in\:{C}_{g}}{d}^{2}\left({x}_{i},{\mu\:}_{g}\right)}$$

where *FI* is the Fisher information, *N*_*g*_ is the number of samples in the cluster *g*, $\:{d}^{2}\left({\mu\:}_{g},\stackrel{-}{X}\right)$ is the square of the distance between the mean of the variable within cluster *g* and the mean of all data for the variable, *C*_*g*_ is the cluster *g*, and $\:{d}^{2}\left({x}_{i},{\mu\:}_{g}\right)$ is the square of the distance between sample *i* and the mean of the variable within cluster *g*^26^. The larger the Fisher information, the greater the contribution of the variable to the clustering.

## Results

**Fig. 2:**
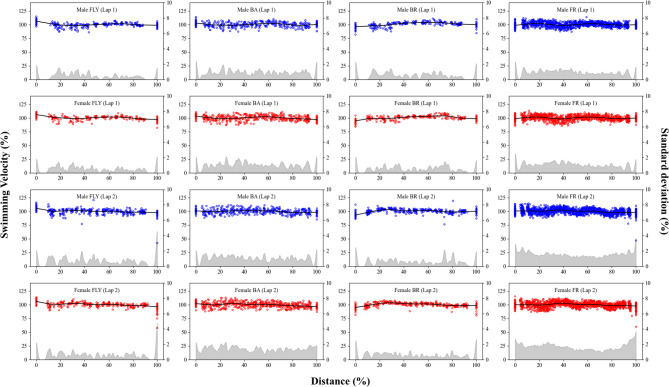
Inter-individual mean trend and the variability for swimming velocity.

**Fig. 3:**
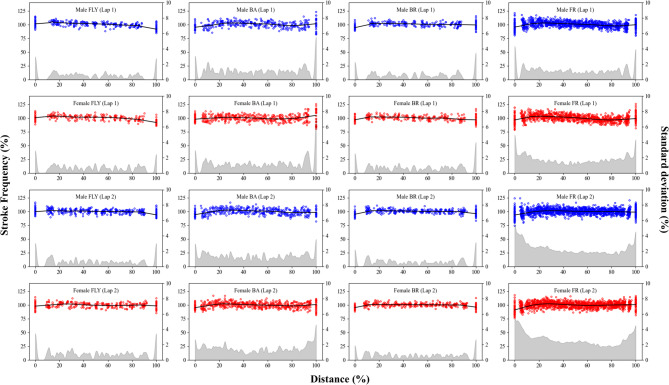
Inter-individual mean trend and the variability for stroke frequency.

**Fig. 4:**
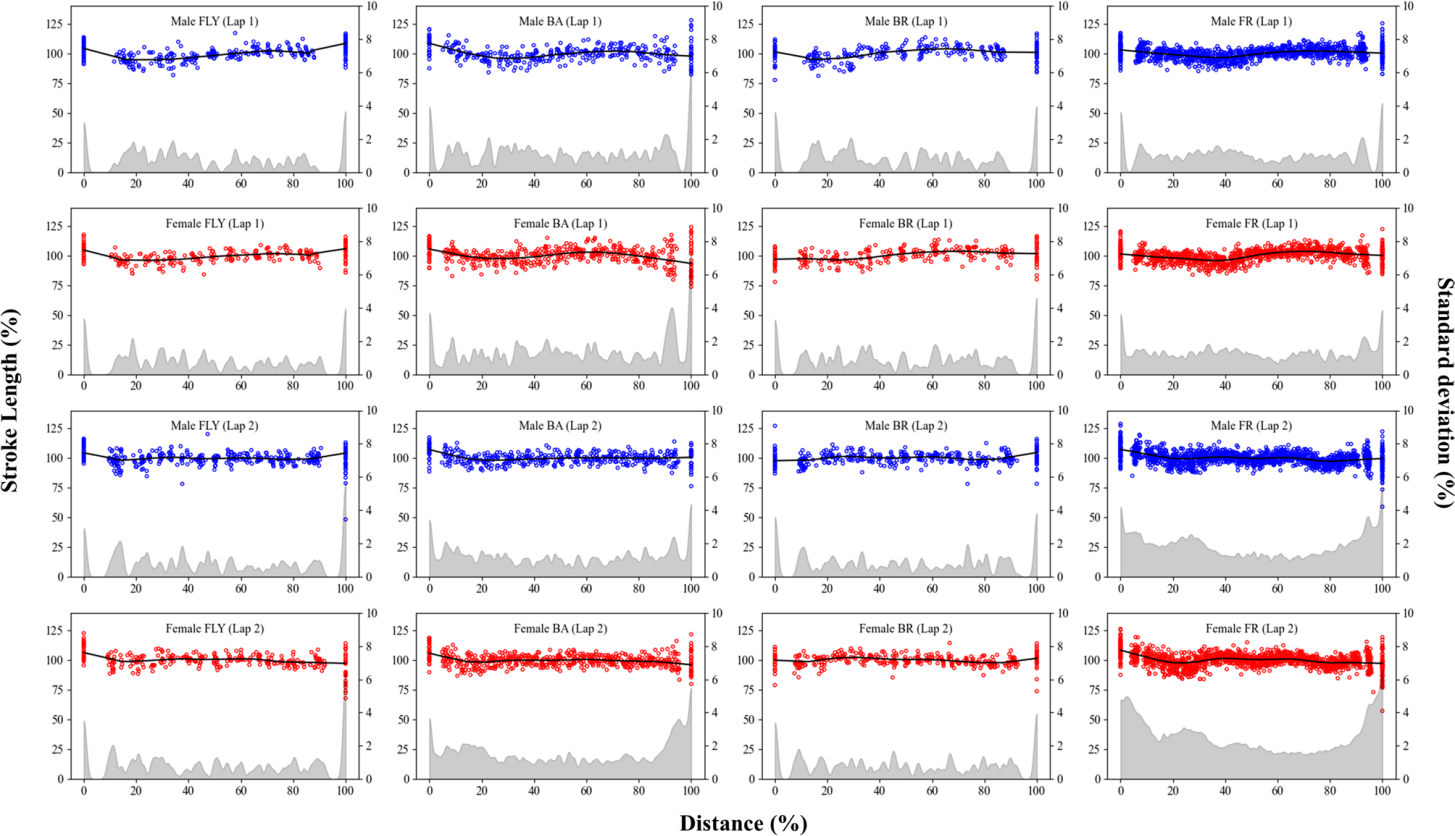
Inter-individual mean trend and the variability for stroke length.

The inter-individual mean trends and the variability for *SF*, *SL*, and *SV* are presented in Figs. 2, 3, and 4, respectively. Swimmers started the laps with *SV* faster than 100% (i.e. intra-lap mean) in butterfly and backstroke, but this trend was not observed in breaststroke and front crawl. Swimmers generally started the lap with *SL* higher than 100% in front butterfly, backstroke, and front crawl, while this trend was less clear in breaststroke. Swimmers exhibited *SF* lower than 100% in the first stroke, with the exception of butterfly where this trend was less clear. For both *SF* and *SL*, the inter-participant variability was large at the beginning and the end of both laps. Particularly, the large variability was evident at 0% (first stroke) and 100% (last stroke) points. Even though the same trend was observed in either/both the beginning or/and the end of many of the *SV* data, it was less evident compared with *SF* and *SL*. When comparing the two laps, in front crawl and butterfly, the swimmers showed greater variabilities in *SV* in lap 2 than in lap 1, particularly at the end of the lap (Fig. 4).

**Fig. 5:**
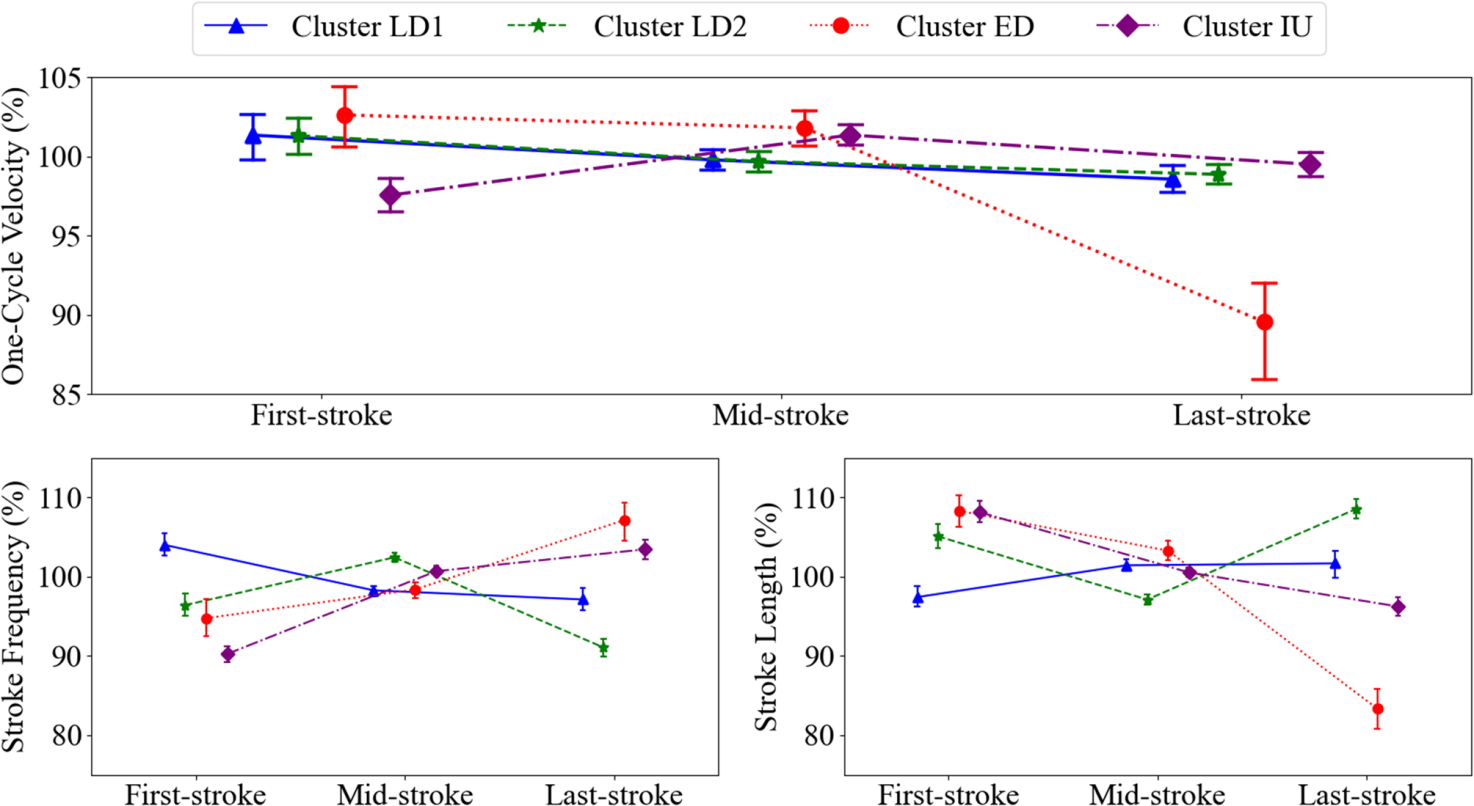
Intra-individual kinematic patterns for the four clusters identified by cluster analysis.

The kinematic strategies of the swimmers in the 50 m time trials were classified into four clusters (Fig. 5). Among the independent variables, the most impactful ones for the clustering were *SF* at the first and last strokes, as well as *SL* at the last stroke. Fisher information for *SF*_*ratio*_, *SL*_*ratio*_, and *SV*_*ratio*_ were all close to zero, meaning that these variables did not have significant impacts on clustering (Table 2). Two clusters showed a linear decrease in *SV* (Cluster LD1 and Cluster LD2). However, the way swimmers achieved this pattern was different between the clusters. Cluster LD1 was characterised by slight increases in *SL* and decreases in *SF*, while Cluster LD2 showed an increase in *SF* and a decrease in *SL* in the mid-stroke, followed by an opposite change (decrease in *SF* and increase in *SL*) in the last stroke. The third cluster had a rather exponential decrease in *SV*, where the mid-stroke was similar to the first stroke, followed by a large decrease in the last stroke (Cluster ED). For this cluster, a similar trend to *SV* was observed in *SL*, whereas *SF* showed the opposite trend (an exponential increase towards the end of the lap). The last cluster exhibited an inverted U-shaped *SV* pattern (Cluster IU), which was characterised by slightly slower *SV* at the first and last stroke compared with the mid-stroke. This trend was accompanied by a constant decrease in *SL* and large and slight increases in *SF.*

**Table 2:** The fisher information for all variables used in the cluster analysis.

	First stroke	Mid-stroke	Last stroke	Ratio
Swimming velocity	0.112	0.074	0.435	0.025
Stroke frequency	0.807	0.329	0.973	0.111
Stroke length	0.455	0.457	1.592	0.090

**Fig. 6:**
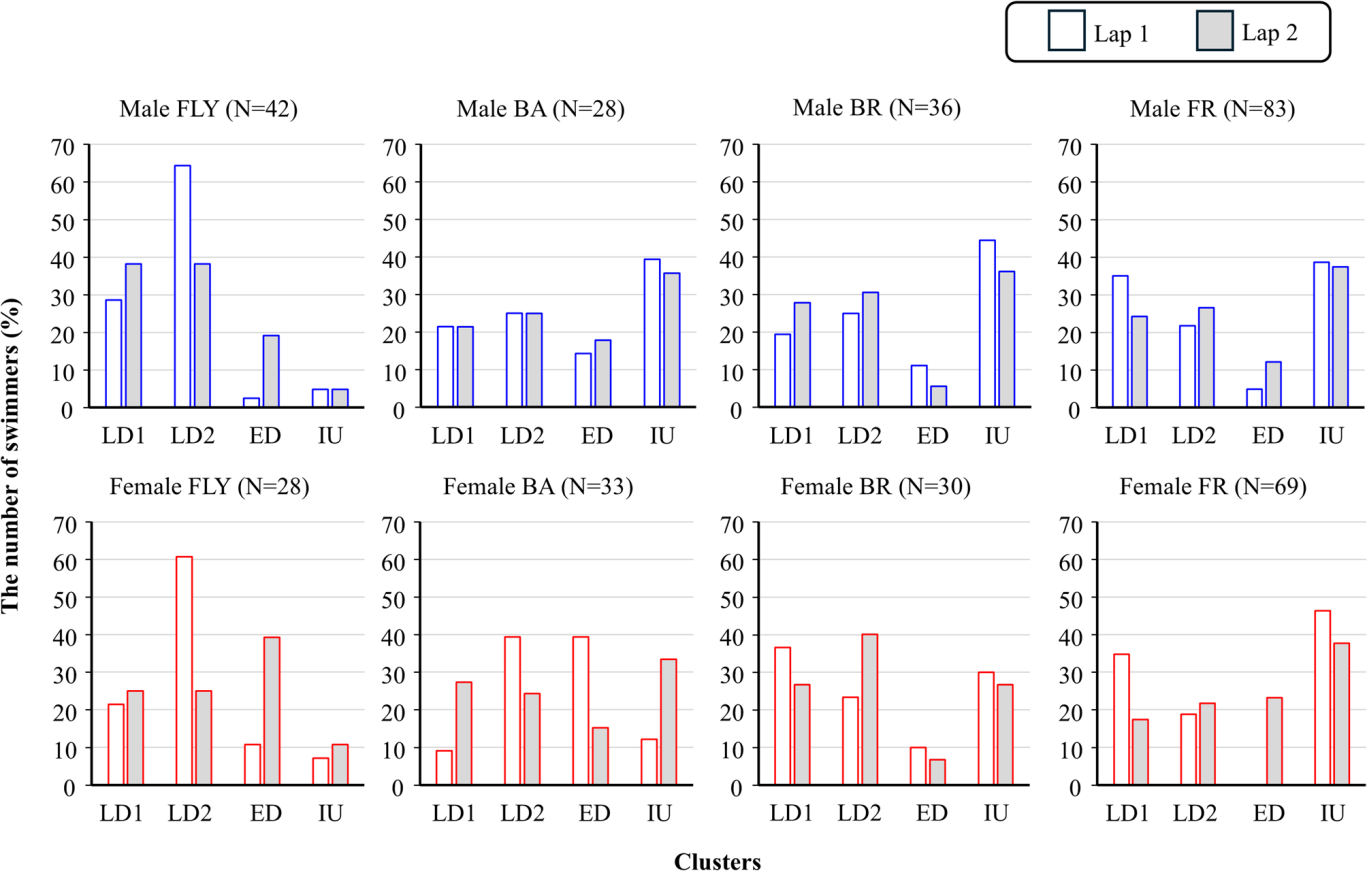
Cluster distribution for each sex, stroke, and lap.

In butterfly, both male and female swimmers showed strong dominancy in Cluster LD2 in lap 1, but the percentage dropped by about 20–40% in lap 2. On the contrary, the percentage of Cluster ED increased by about 15–30% (Fig. 6). Cluster IU was the least common pattern in the butterfly for both sexes and laps. The opposite trend was observed in front crawl and backstroke, where Cluster IU was the most common pattern, except for lap 1 in female swimmers for backstroke. In breaststroke, Cluster IU was the most dominant pattern for males, while Cluster LD1 and LD2 (for lap 1 and lap 2, respectively) were dominant in female swimmers. For both sexes, Cluster ED was the least dominant pattern in breaststroke. In female swimmers, the change in cluster distribution among the participants was more noticeable, particularly in the backstroke (Fig. 6).

## Discussion

The present study was the first attempt to investigate the inter- and intra-individual variabilities of competitive swimmers in kinematic parameters (*SF*, *SL*, and *SV*) for all four strokes. The main outcomes were that swimmers generally showed a large variability at the beginning and the end of the lap, and intra-individual variabilities are classified as four different patterns, primarily based on how the first and/or last strokes differ from the mid-stroke. The descriptive results of the inter-individual variability were aligned with the initial hypothesis and provided important insights into race analysis. Currently, it is common to divide a race into the start, clean-swimming, turn, and finish segments, but the definitions of those segments vary among studies. Particularly, there have been seven different definitions for the turn segment^4^ with the turn-in phase (swimming towards the wall) ranging from 7.5 m to 0 m^5,9,27^. The inter-individual standard deviations in *SF* and *SL* were generally higher at both ends of each lap (the first and last stroke), showing that individual strategies of swimmers adjusting their movements around the breakout and towards the turn were particularly evident. In the present study, the 100% distance point (where the swimmers started their last strokes) was in the range of 21.5–22.8 m, with a standard deviation of about 0.4–0.9 m. According to the empirical rule (95% of the data will fall within two standard deviations in normally distributed data), this means that 95% of swimmers likely start the last stroke around 20 m point at the earliest. Therefore, setting the start of the turn-in phase as 5 m before the wall is reasonable, as has been done in many studies^28–30^. Similarly, the large variety at the end of lap 2 also justifies the need to separate the last part of the race. The 100% point was around 21.9–22.9 m with a standard deviation of 0.4–0.7 m, which again implies that the earliest point swimmers start the last stroke is around 5 m before the wall, and therefore, setting this range as the finish phase^8,28,31^ is also reasonable. Nonetheless, given that the 95% confidence range for the start of the last stroke could be as large as 1.5–3.5 m, defining those phases based on the individual timing of the start of the last stroke would be preferable, as suggested in the literature^5^.

A previous study has suggested that the first stroke (transition stroke), particularly after the start, should be treated with caution in race analysis because the kinematic variables are generally superior in this stroke compared with the rest^10^. The present study supports this suggestion from the perspective of inter-participant variability. Similar to the last stroke, the first stroke also showed greater variability than mid-lap strokes, particularly in *SF* and *SL*, meaning that how swimmers switch underwater to free swimming segments considerably varied. There are several potential constraints that affect the variabilities, such as the duration and forward velocity of the underwater segment, the need to elevate the body from underwater to the water surface, and the start of the upper limb propulsive movements^11,32^. There have been few studies on race analysis which account for the effect of the first stroke. Interestingly, some studies in the 1980s defined the end of the start and turn segments as the point where the swimmer completes the first arm cycle after the breakout^33,34^ but later race analysis studies did not follow this definition, and instead, either the 15 m point^9^ or the head breakout point^5^ has been a common key event in defining the start and turn segments. This might be due to the introduction of the ‘15 m rule’ by World Aquatics in the 1990s, which probably stressed the importance of both the 15 m point and the location of the head breakout. However, the present results suggest that race analysts should focus on this specific additional segment (e.g. from the head breakout until the end of the first stroke) to better understand each swimmer’s transition technique from the underwater to subsequent mid-pool surface swimming segment.

Among the four identified clusters for the intra-individual variability and their occurrence by strokes, Cluster IU was characterised by the slower *SV* in the first stroke compared with the rest. This pattern accounted for about 7%, 30%, 34%, and 40% of butterfly, backstroke, breaststroke, and front crawl swimmers, respectively, which was not aligned with the second initial hypothesis (the majority of swimmers would show the fastest velocity at the beginning). This pattern is likely not preferable for many strokes as it demonstrates that swimmers in this Cluster either propelled underwater for too long or lost momentum from the start/turn during the transition. The only exception is breaststroke, where a significant decrease in velocity could be expected at the end of the underwater segment^35^. Interestingly, Cluster IU was the least common pattern in butterfly, whereas it was the most common pattern in front crawl for both lap 1 and lap 2. This difference might be due to a distinct task complexity in these two strokes. In butterfly, both underwater and surface swimming are characterised by in-phase movements of the left and right limbs and the undulatory movement of the body, implying that swimmers could start the first stroke as a sequential movement from underwater undulatory swimming. However, this is not the case in front crawl swimming transition, where swimmers are required to switch from the in-phase and undulatory movements to the anti-phase movements of the left and right limbs. As in-phase and anti-phase have different rhythm control mechanisms^36^ and in-phase movements are more stable than anti-phase^37^the transition in front crawl is probably more complex than that in butterfly, which might be a reason for many front crawl swimmers exhibiting the Cluster IU pattern. This could also explain why a greater percentage of backstrokers performed with the Cluster IU pattern compared to butterfly swimmers. In fact, for both laps and sexes, 60–70% of butterfly swimmers showed either Cluster LD2 or Cluster ED, which both included a higher *SV* in the first stroke than in the mid-stroke due to a longer *SL*, despite a lower *SF*. These data further emphasised that these swimmers proficiently transferred the underwater swimming momentum to the surface swimming and are in line with previous reports of butterfly stroke presenting the fastest forward velocities in the first stroke^11^despite overall front crawl race times being faster. Regarding the inter-individual variability, another important aspect is that by comparing the general trend lines displayed in Fig. 4 and the cluster distribution exhibited in Fig. 6, it is clear that the general trend is not necessarily a good indicator of how the majority of swimmers behave within the lap. For example, in breaststroke, the general trend shows a slower velocity at the beginning of the lap compared with the subsequent clean swimming. However, breaststroke swimmers who were grouped into Clsuter IU (which was the only cluster that had a slower velocity in the first stroke compared to the mid-stroke) were less than 50% of the samples.

It is worth mentioning that the number of swimmers exhibiting the Cluster ED pattern greatly increased in lap 2 compared with lap 1 in both front crawl and butterfly. This cluster pattern shows a very large drop in *SV* in the last stroke due to a large decrease in *SL*, despite a noticeable increase in *SF*. The increase in this pattern can explain why the inter-participant variability at the end of the lap was larger in lap 2 compared with lap 1 in these two strokes, and it could be related to the fatigue and deterioration in mechanical efficiency at the end of 50 m races^17^. Conversely, in breaststroke, Cluster ED was the least common pattern in both lap 1 and lap 2, showing that many swimmers did not greatly drop their *SV* at the end of the lap. This result also indicates that it is uncommon for breaststrokers to increase *SF* (and decrease *SL*) to adjust their stroke movement for the wall touch. In backstroke, female swimmers had noticeable differences in the cluster distribution between lap 1 and lap 2. In lap 1, Cluster LD2 and Cluster ED (characterised by a large increase/decrease in *SF* and *SL* at the last stroke) were dominant with each cluster accounting for 40% of the participants. This might suggest that female swimmers tend to lose their *SV* to a greater extent (compared with males) due to the adjustment of *SL* and *SF* when rolling the body from the supine to the prone position for the tumble turn.

Some limitations of the current study should be noted. Firstly, the current study was conducted in an observational manner to establish inter- and intra-individual variabilities in kinematic parameters (*SF*, *SL*, and *SV*). However, the results of the present study cannot explain the mechanisms behind the variabilities. Therefore, future studies employing experimental settings, focusing on the transition from underwater to surface swimming as well as the end of each lap, would be useful. Secondly, similarities in the cluster distribution between the two laps observed in many cases do not necessarily mean that swimmers did not change their cluster patterns from lap 1 to lap 2, as the current study did not analyse individual consistencies from lap 1 to lap 2 (the metrics show the overview of intra-lap variability patterns and do not reflect individual inter-lap variability). For a better understanding of inter-lap variabilities or across multiple trials, testing the intra-individual consistency of the cluster patterns in a repeated-measures design will be helpful in future studies. Lastly, the present study utilised an automatic race analysis system, which generated a somewhat limited number of kinematic variables (*SF*, *SL*, and *SV*) compared with more detailed motion analysis methods. However, it should be emphasised that biomechanical motion analysis approaches have a limited capture volume and often require long data processing/analysis time, which makes it highly challenging to focus on the stroke-to-stroke variability topics. Due to a recent change in the WA regulation, swimmers are now allowed to use approved wearable devices in competitions^38^. Thus, there will likely be more studies in the future on stroke-to-stroke variability during the race, for which the methods and outcomes from the present study will be of fundamental insight.

## Conclusions

In short course 50 m swimming races, swimmers show large inter-individual kinematic variabilities at the beginning and the end of the lap. Therefore, researchers and performance analysts should note that, when possible, the first and last strokes should be separated from the clean-swimming segment in race analysis as opposed to the traditional start-swimming-turn race division. Overall, intra-lap kinematic patterns of each stroke and sex can be described by four clusters depending on how *SV*, *SF*, and *SL* change throughout the lap. The distribution of clusters among the swimmers and laps indicated that it is likely more difficult for alternating strokes (front crawl and backstroke) than butterfly swimming to transfer the momentum from underwater swimming to surface swimming. This information is particularly important for swimmers and coaches as it implies that front crawl and backstroke swimmers, in comparison to butterfly swimmers, probably should invest more time and effort in improving transition technique. In addition, the different technical constraints at the end of each race lap (turn or finish segment) likely affect the management of *SF* and *SL*, leading to large kinematic variabilities. Overall, swimming kinematics should be examined not only by focusing on the general trend but also by considering individual variabilities to successfully capture the swimmers’ behaviour and technical proficiency, as well as to provide a clear rationale for the definitions of different race segments.

## Data Availability

The datasets used and/or analysed during the current study are available from the authors upon reasonable request.
